# Pediatric Musculoskeletal Radiograph Interpretation Among Orthopaedic Surgery Postgraduate Trainees: A Competency-Based Education Intervention

**DOI:** 10.1097/BPO.0000000000003446

**Published:** 2026-08-19

**Authors:** Carl A. Starvaggi, Maryse Bouchard, Mark Camp, Sasha Carsen, Alexandra J. Mortimer, Ryan Katchky, Ron El-Hawary, Lori A. Archer, Neil Saran, Jennifer Stimec, Kai Ziebarth, Kristina Keitel, Kathy Boutis

**Affiliations:** * Graduate School for Health Sciences, University of Bern, Bern, Switzerland; † Department of Pediatric Surgery, Inselspital, Bern University Hospital, University of Bern, Bern, Switzerland; ‡ Department of Orthopedics, Spitalzentrum Oberwallis, Brig, Switzerland; § Division of Orthopedic Surgery, Department of Surgery, Hospital for Sick Children and University of Toronto, Toronto, Ontario, Canada; ‖ Division of Orthopedic Surgery, University of Toronto, Toronto, Ontario, Canada; ¶ University of Saskatchewan, Jim Pattison Children's Hospital, Saskatoon, Saskatchewan, Canada; # Scarborough Health Network, Scarborough, Ontario, Canada; ** Division of Orthopedic Surgery, IWK Health Centre, Halifax, Nova Scotia, Canada; †† Section of Orthopedic Surgery, University of Manitoba, Winnipeg, Manitoba, Canada; ‡‡ Division of Orthopedic Surgery, Department of Pediatric Surgery, McGill University, Montreal, Quebec, Canada; §§ Department of Diagnostic and Interventional Radiology, Hospital for Sick Children and University of Toronto, Toronto, Ontario, Canada; ‖‖ Laboratory for intelligent Global Health and Humanitarian Response (LiGHT), EPFL, Lausanne, Switzerland; ¶¶ Division of Pediatric Emergency Medicine, Department of Pediatrics, Hospital for Sick Children and University of Toronto, Toronto, Ontario Canada

**Keywords:** traumatology, radiographs, musculoskeletal imaging, competency-based medical education, residency, training

## Abstract

**Background:**

Pediatric musculoskeletal radiographs (pMSK) are the primary imaging modality for evaluating injured children, yet there is no widely adopted, standardized approach to teach and assess this specific skill among orthopaedic surgery postgraduate trainees (OS-PGT). We evaluated an online educational and assessment intervention using pMSK radiographs to improve fracture/dislocation detection and risk stratification among OS-PGT.

**Methods:**

We conducted a multicenter prospective cohort study using a web-based educational intervention, recruiting a convenience sample of OS-PGT. The intervention included 1609 pMSK radiograph cases organized into 6 body regions. OS-PGT judged whether a fracture/dislocation was present or absent, and when present, localized the abnormality and assigned a risk category. Participants received immediate feedback after every case, continuing until reaching a predefined 90% performance standard. Following the intervention period, participants were invited to complete a post-intervention survey.

**Results:**

A total of 64,489 unique case interpretations were generated by 115 OS-PGT. From initial to final 25 cases completed, diagnostic accuracy increased by 10.5% (95% CI: 9.4%, 11.6%), localization accuracy by 11.9% (95% CI: 10.7%, 13.0%), and risk stratification accuracy by 20.5% (95% CI: 18.9%, 22.1%), with the greatest gains observed for ankle/foot radiographs. Across 246 attempts to reach the performance standard, 192 (78.0%) were successful. The median number of cases required per body region was 141 (IQR: 96, 313), completed in a median of 34.9 minutes (IQR: 23.8, 77.6). Error rates were lower when participants were certain of their answers (7.8% incorrect “definite” interpretations vs. 23.7% “probable”). Forty-five (39.1%) participants completed the post-intervention survey, and 38 (84.6%) supported integrating the intervention into orthopaedic residency training.

**Conclusion:**

This study shows that a structured, web-based intervention combining performance-based assessment with case-based practice led to substantial and efficient learning gains in pMSK radiograph interpretation among OS-PGT and helped identify diagnostic challenges. These findings support the use of such educational interventions as a practical strategy to supplement orthopaedic residency training in targeted diagnostic skills.

**Levels of Evidence:**

Level II—prospective cohort study.

Musculoskeletal injuries are common among children and adolescents, accounting for more than 11 million health care visits annually in the United States alone,^1,2^ and radiographs are the principal imaging modality in orthopaedic practice to identify musculoskeletal injuries.^3,4^ However, pediatric musculoskeletal (pMSK) radiographs can be difficult to interpret because of age-dependent skeletal maturation and unique pediatric fracture patterns.^5,6^ Consequently, they are vulnerable to diagnostic error, particularly among less experienced interpreters.^7^ Although the number of pediatric orthopaedic subspecialists has increased, approximately half of children with musculoskeletal injuries are treated by general orthopaedic surgeons.^8,9^ While most missed injuries are subtle fractures with limited clinical consequences,^10,11^ delayed or missed high-risk fractures may result in growth disturbance, deformity, and long-term functional impairment.^6,12^ Although official radiology reports are provided by radiologists, treating orthopaedic surgeons are frequently the first to interpret pMSK radiographs in emergency settings and must make immediate treatment decisions based on their own readings, particularly when official reports are delayed during after-hours or weekend coverage.^13–15^ Given the high volume of pMSK radiographs, even a small proportion of missed high-risk injuries can lead to substantial morbidity, increased health care utilization, and medicolegal risk.^16,17^

Despite the importance of accurate pMSK radiograph interpretation, there is no widely adopted, standardized approach specifically designed to teach and assess this skill among orthopaedic surgery postgraduate trainees (OS-PGT). Many medical students applying to orthopaedic residency feel unprepared to interpret musculoskeletal radiographs and report a need for formal instruction,^18,19^ with early residents stating that a basic skills course in reading radiographs would have improved their performance during the first 2 years of residency. Although orthopaedic residency programs often include structured weekly didactic teaching and often incorporate online educational resources,^20^ these approaches are generally broad in scope and are not designed for focused simulation of a single diagnostic skill.^21,22^ The development of specific skills, therefore, often relies on bedside teaching and opportunistic case exposure, which may result in knowledge gaps due to variability in clinical encounters and training environments.^23,24^ Pediatric orthopaedic exposure also varies across residency programs in duration and structure.^25^ Training frequently lacks structured feedback and opportunities for deliberate practice or self-reflection,^26^ limiting consistency across programs. Although competency-based medical education (CBME) emphasizes explicit standards and evidence of competence,^27^ no evidence-based method currently exists to support deliberate skill development and performance assessment in pMSK radiograph interpretation among OS-PGT.^28^

The primary objective of this study was to determine the effectiveness of a structured educational and assessment intervention in improving OS-PGT accuracy in pMSK radiograph interpretation, specifically in fracture/dislocation detection and risk stratification. Secondary objectives were to assess the learning effort required to reach a predefined performance standard, evaluate diagnostic certainty, and describe participant feedback.

## METHODS

### Study Design and Setting

This multicenter prospective cohort study evaluated an online educational intervention. The study was approved by the Institutional Review Board at the Hospital for Sick Children, Toronto, Ontario, Canada.

### Educational Intervention Development

#### Case Acquisition and Diagnostic Composition

Development of the educational and assessment intervention was grounded in education theory and has been described previously.^24^ Our group developed an online learning tool containing 1609 pediatric musculoskeletal (pMSK) cases for fracture/dislocation detection and risk stratification. Images were sourced from the lead site and, for rarer but clinically important cases, from a Canadian pediatric orthopaedic surgeon collaborative network.^24^ Cases were organized into 6 body regions: shoulder/clavicle/humerus; elbow; forearm/wrist/hand; pelvis/femur; knee/tibia-fibula; and ankle/foot (∼200 to 350 cases per region; Table 1).

**TABLE 1:** Case Characteristics

	All regions (N=1609)	Clavicle/shoulder/humerus (N=230)	Elbow (N=343)	Forearm/wrist/hand (N=282)	Pelvis/femur (N=205)	Knee/tibia-fibula (N=245)	Ankle/foot (N=304)
Age, mean±SD	8.1±4.6	7.6±4.9	6.9±3.9	8.8±4.4	7.8±4.9	6.7±5.1	10.3±4.0
Male, no. (%)	919 (57.1)	143 (62.2)	169 (49.3)	173 (61.3)	123 (60.0)	141 (57.6)	170 (55.9)
Fracture/dislocation present, no. (%)	1093 (67.9)	153 (66.5)	243 (70.8)	182 (64.5)	143 (69.8)	159 (64.9)	213 (70.1)
Risk, no. (%)							
Low	173/1093 (15.8)	69/153 (45.1)	—^*^	21/182 (11.5)	2/143 (1.4)	22/159 (13.8)	59/213 (27.7)
Medium	366/1093 (33.5)	53/153 (34.6)	84/243 (34.6)	83/182 (45.6)	5/143 (3.5)	77/159 (48.4)	64/213 (30.0)
High	554/1093 (50.7)	31/153 (20.2)	159/243 (65.4)	78/182 (42.9)	136/143 (95.1)	60/159 (37.7)	90/213 (42.3)
High suspicion of nonaccidental trauma, no. (%)	25/1093 (2.3)	5/153 (2.2)	1/243 (0.3)	0	11/143 (5.4)	6/159 (2.4)	2/213 (0.7)

Reprinted from *Academic Radiology*; Boutis K, Starvaggi C, Doria AS, Bouchard M, Camp M, Taylor J, Hauge CJ, Carter O, Stimec J; Optimizing Radiology Resident Competency in Pediatric Musculoskeletal Radiograph Interpretation; Epub ahead of print; Copyright (2026), with permission from Elsevier.

^*^ There were no low-risk elbow fractures or dislocations in the case set. All elbow injuries were classified as medium- or high-risk according to the risk stratification definition framework.

The case mix spanned the pediatric age spectrum (0 to 17 y; mean 8.1±4.6 y) and included a spectrum of cases with and without fractures/dislocations.^29,30^ Because high diagnostic sensitivity is a priority in pMSK interpretation, 67.9% of cases contained a fracture/dislocation.^24,31^ Each fracture was categorized by location, morphology, and degree of displacement to determine the most appropriate management, which defined risk stratification. Low risk was defined as cases that could be managed with symptom-guided immobilization and primary physician follow-up. Medium risk cases were those that would benefit from rigid immobilization and orthopaedic surgery follow-up, while high risk cases required urgent manipulation, open reduction/fixation, or were suspicious for a nonaccidental injury.^10,24^ The final case-mix included 15.8% low-risk, 33.5% medium-risk, and 50.7% high-risk fractures/dislocations (Table 1).

Radiographic diagnoses and risk categories were initially verified by the official radiology report, a pediatric emergency physician, an orthopaedic surgery resident, and a radiology resident.^24^ Cases with any degree of diagnostic or management uncertainty were also reviewed by 2 pediatric orthopaedic surgeons and a subspecialized pMSK radiologist, who established the final diagnosis and risk category.^24^

#### User Design

Cases were presented on an online learning platform with standardized case histories and corresponding radiographs (Fig. 1, video demo, Supplemental Digital Content 1, https://links.lww.com/BPO/B121).^24^ Case histories were limited to one sentence containing only age, sex, and the primary reason for presentation to focus learning in radiograph interpretation and minimize any bias from clinical context.^32^ Participants indicated whether a fracture/dislocation was “definitely present,” “probably present,” “probably absent,” or “definitely absent,” allowing assessment of diagnostic certainty.^33^ If a fracture/dislocation was suspected, participants localized it and assigned a risk category.^24^ Immediate text and visual feedback were provided after each submission. Cases were presented in a unique order for each participant to balance difficulty and avoid sequential context effects.^34^ Performance summaries were generated after approximately 58 cases once participants had completed a minimum of 25 fracture-positive and 25 fracture-negative cases. Participants continued until reaching a predefined performance standard of 90% accuracy (overall accuracy), 90% sensitivity (fracture/dislocation positive cases), and 90% specificity (fracture/dislocation negative cases).^24^ This threshold exceeded the previously suggested 85% benchmark for emergency physicians because orthopaedic trainees make final treatment decisions in acute settings.^10^ The permitted 10% error rate considers that the fractures most commonly missed are subtle, low-risk fractures with limited clinical consequences.^10,11^ All platform interactions were logged for learning analytics.^35^ The intervention was pilot tested by 5 orthopaedic surgeons and 5 radiologists external to the study team.^24^

**FIGURE 1:**
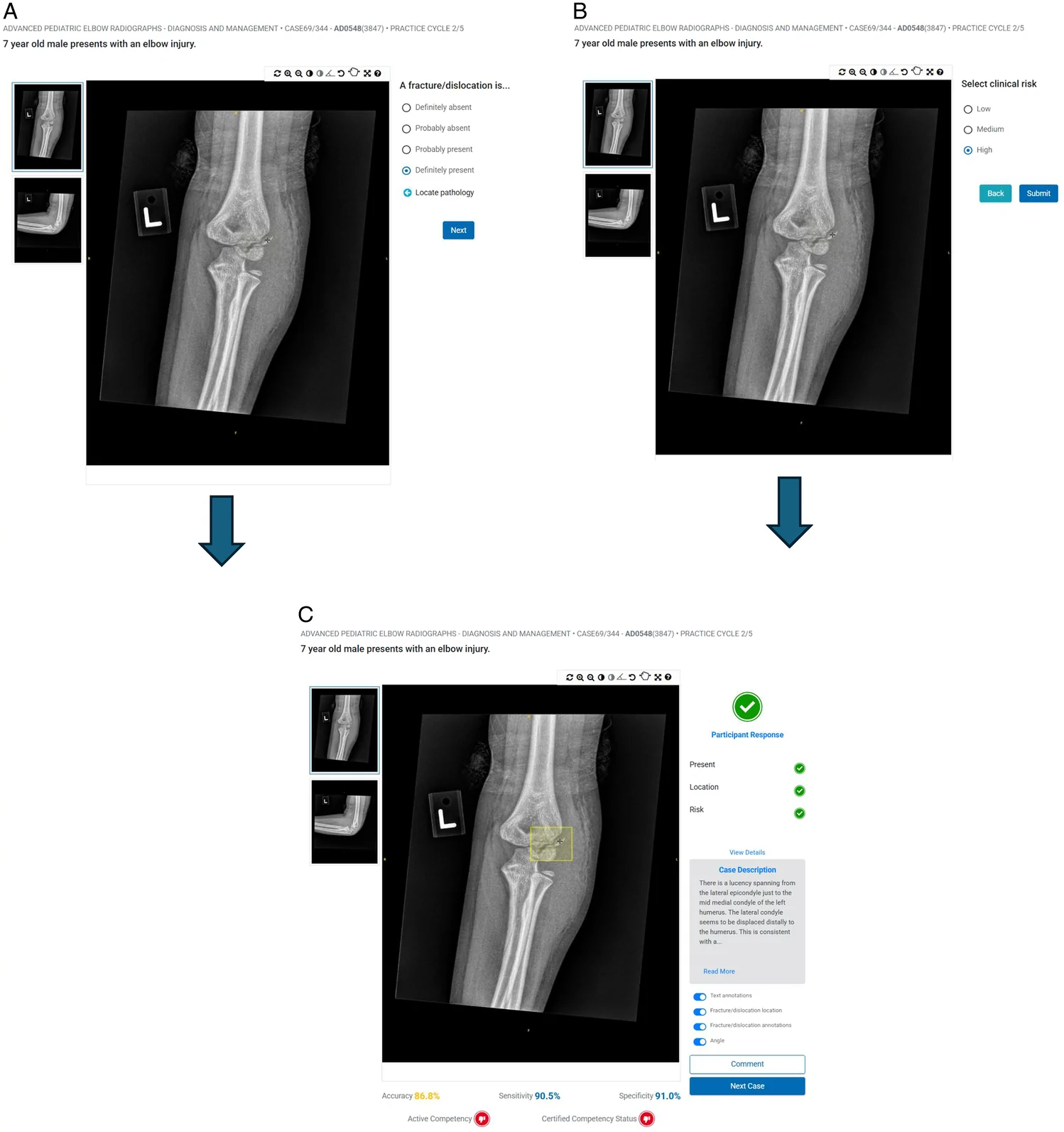
Participants are asked if there is a fracture/dislocation present or absent (A). If they select “present,” they must locate the fracture (A) and then risk-stratify the abnormality (B). After they submit their response, they get feedback after every case (C). After 25 cases with and 25 cases without a fracture/dislocation (average of 58 cases total), they get their diagnostic accuracy (overall performance), sensitivity (cases with a fracture/dislocation), and specificity (cases without a fracture/dislocation). The performance standard was reached when participants achieved 90% accuracy, sensitivity, and specificity.

### Participants

We enrolled a convenience sample of OS-PGT training in Canada and Switzerland. Participants were included in the analysis if they completed at least 50 cases in at least one body region. In Canada, recruitment occurred through residency program directors using an email invitation distributed through Mailchimp (Intuit Inc., Atlanta, GA, USA), with 5 solicitations sent during the recruitment period. In Switzerland, department heads responsible for residency training were contacted directly and asked to present the project to residents before distributing the registration link. Nonresponding departments were contacted again by email or in person at conferences.

### Data Collection and Postintervention Survey

Participants provided consent upon registration and received login credentials. Collected demographics included sex, geographic location, and training year. There was no time limit per case, and the platform remained accessible throughout the study. Participants had 12 weeks to reach the performance standard in at least one body region before their account was closed. Reminder emails were sent every 2 weeks. Two weeks after account closure, all registered participants received a link to a post-intervention survey assessing preferred performance standards, perceived relevance of the intervention, and barriers to completion. Up to 3 solicitation emails were sent for the follow-up survey. Participants achieving the performance standard across all 6 regions within the 12-week period received a gift card.

### Outcomes and Analyses

#### Overall

The primary outcome was the change in diagnostic accuracy. Secondary outcomes included changes in sensitivity and specificity, the number of cases required to reach the performance standard, participant certainty, and participant feedback.

#### Scoring and Performance Metrics

Each case contributed a maximum of one point. For fracture-negative cases, one point was awarded for correctly identifying the absence of injury. For fracture-positive cases, 0.5 points were awarded for correct detection, 0.25 for accurate localization, and 0.25 for correct risk stratification. Certainty ratings were not incorporated into scoring.

To estimate within-participant learning, diagnostic performance on the first 25 and last 25 cases was compared.^36^ Metrics included overall accuracy (total correct/all responses); diagnostic sensitivity [true positive fracture/(true positive fracture+false negative fracture)]; diagnostic specificity [true negative fracture/(true negative fracture+false positive fracture)]; case review time; localization accuracy; and risk-stratification accuracy. To assess the educational impact, we calculated Cohen's effect sizes.^37^ Changes in performance metrics and effect sizes were presented with their respective 95% CIs. Performance changes across body regions were compared using the Kruskal-Wallis test.

#### Cases Completed to Reach the Performance Standard

We presented the number of attempts to reach the performance standard within each body region, as well as the number of cases required to do so. An attempt was defined as a body region started by a participant in which at least one case was completed. Because case counts were skewed, the number of cases required to reach the performance standard was summarized using medians and interquartile ranges. Differences across body regions were evaluated using the Kruskal-Wallis test. Multilevel logistic regression assessed whether achieving the performance standard was associated with postgraduate year (PGY) (≤2 vs. ≥3) or country (Canada vs. Switzerland).

#### Participant Certainty

We summarized the proportion of participants assigning “definite” responses in the initial versus final 25 cases and tested the change using a paired Student's *t* test. We reported the frequency of “definite” and “probable” certainty assignments and the corresponding proportion of incorrect interpretations, including false negatives and false positives.

#### Participant Feedback

We reported the frequency of preferred performance standards selected by participants, and the proportion of participants who agreed/strongly agreed with each item on the postintervention survey.

#### Sample Size Considerations

Sample size was determined for a paired pre-post comparison of diagnostic accuracy using the McNemar test, assuming a discordant pair proportion of 0.15.^5^ Under these assumptions, 115 participants were required to detect a 10% absolute increase in accuracy with 80% power at a 2-sided α of 0.05 (PASS 11, Kaysville, UT, USA).

Multiple testing was addressed using a Bonferroni adjustment when relevant. Statistical analyses were conducted in SPSS Statistics (version 29; IBM Corp, Armonk, NY, USA).

## RESULTS

### Study Population

Between March 1 and September 30, 2024, 136 OS-PGT registered to participate. Of these, 28 (20.6%) engaged in cases in one region only, while 66 (48.5%) participated in all 6, and 115 (84.6%) OS-PGT completed at least 50 cases in at least one body region (Fig. 2).

**FIGURE 2:**
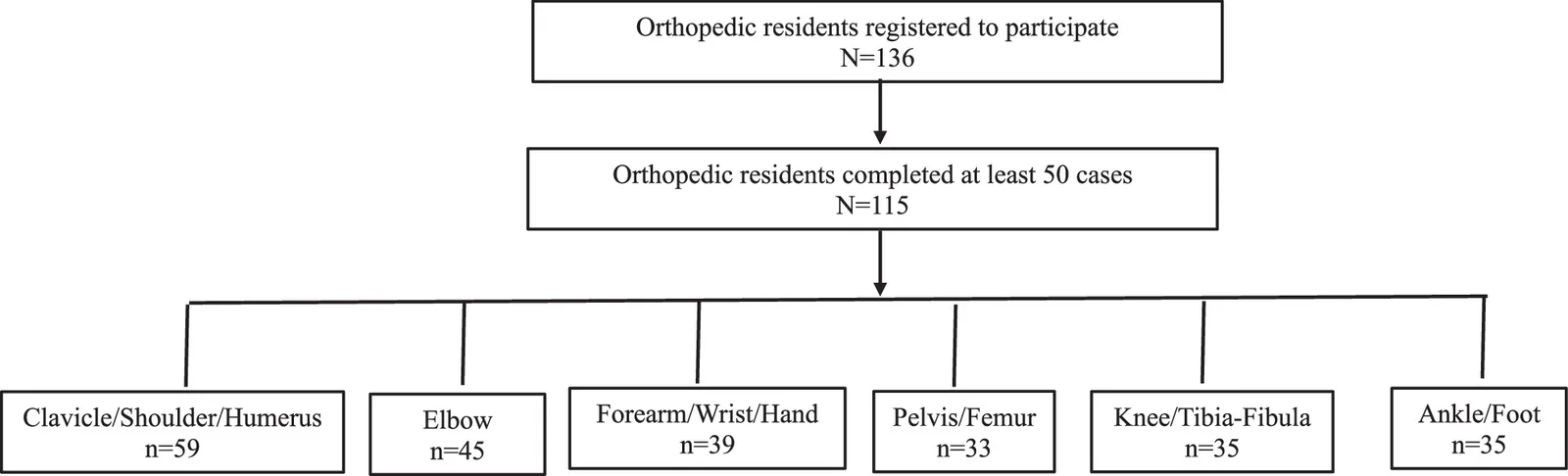
Participant engagement.

In the latter cohort, 64 (55.7%) identified as male, and 63 (54.8%) were in PGY 1 to 2, 30 (26.1%) in PGY 3 to 4, and 22 (19.1%) in PGY ≥5. Sixty-one (53.0%) trained in Switzerland and 54 (47.0%) in Canada. Participants generated 64,489 unique case interpretations across 1609 cases, with a median of 196 (IQR: 104-315; min 58, max 1715) cases completed per participant.

### Diagnostic Performance

Diagnostic accuracy increased significantly by +10.5% (95% CI 9.4%, 11.6%), diagnostic sensitivity by +13.3% (95% CI 12.1%, 14.6%), and diagnostic specificity by +6.5% (95% CI 5.6%, 7.4%), with the time spent per case decreasing from 66.2 to 18.7 seconds (difference −47.5; 95% CI: −57.9, −38.0) (Table 2). Effect sizes were large across diagnostic performance metrics.^37^ Accuracy gains differed significantly between body regions (*P*<0.001), with post hoc adjusted analyses demonstrating greater improvement in ankle/foot compared with forearm/wrist/hand (*P*=0.01), elbow (*P*=0.01), knee/tibia-fibula (*P*=0.04), and pelvis/femur (*P*<0.001) (Fig. 3A). There was no association of change in accuracy with country (*P*=0.24) or PGY ≥3 versus ≤2 (*P*=0.18).

**TABLE 2:** Changes in Diagnostic Performance Among Orthopaedic Surgery Postgraduate Trainees

Performance parameter	Initial, final N=115	Delta (95% CI) N=115	Cohen's *d* (95% CI) N=115
All case tasks			
Diagnostic accuracy (%)	84.4, 94.9	10.5 (9.4, 11.6)	1.3 (1.1, 1.4)
Diagnostic sensitivity (%)	78.2, 91.6	13.3 (12.1, 14.6)	1.4 (1.2, 1.5)
Diagnostic specificity (%)	88.7, 95.2	6.5 (5.6, 7.4)	0.9 (0.8, 1.1)
Time spent per case (s)	66.2, 18.7	−48.0 (−57.9, −38.0)	−0.6 (−0.7, −0.5)
Location			
Location accuracy (%)	82.8, 94.7	11.9 (10.7, 13.0)	1.3 (1.1, 1.6)
Risk stratification			
Risk stratification accuracy (%)	65.2, 85.7	20.5 (18.9, 22.1)	1.6 (1.4, 1.8)

**FIGURE 3:**
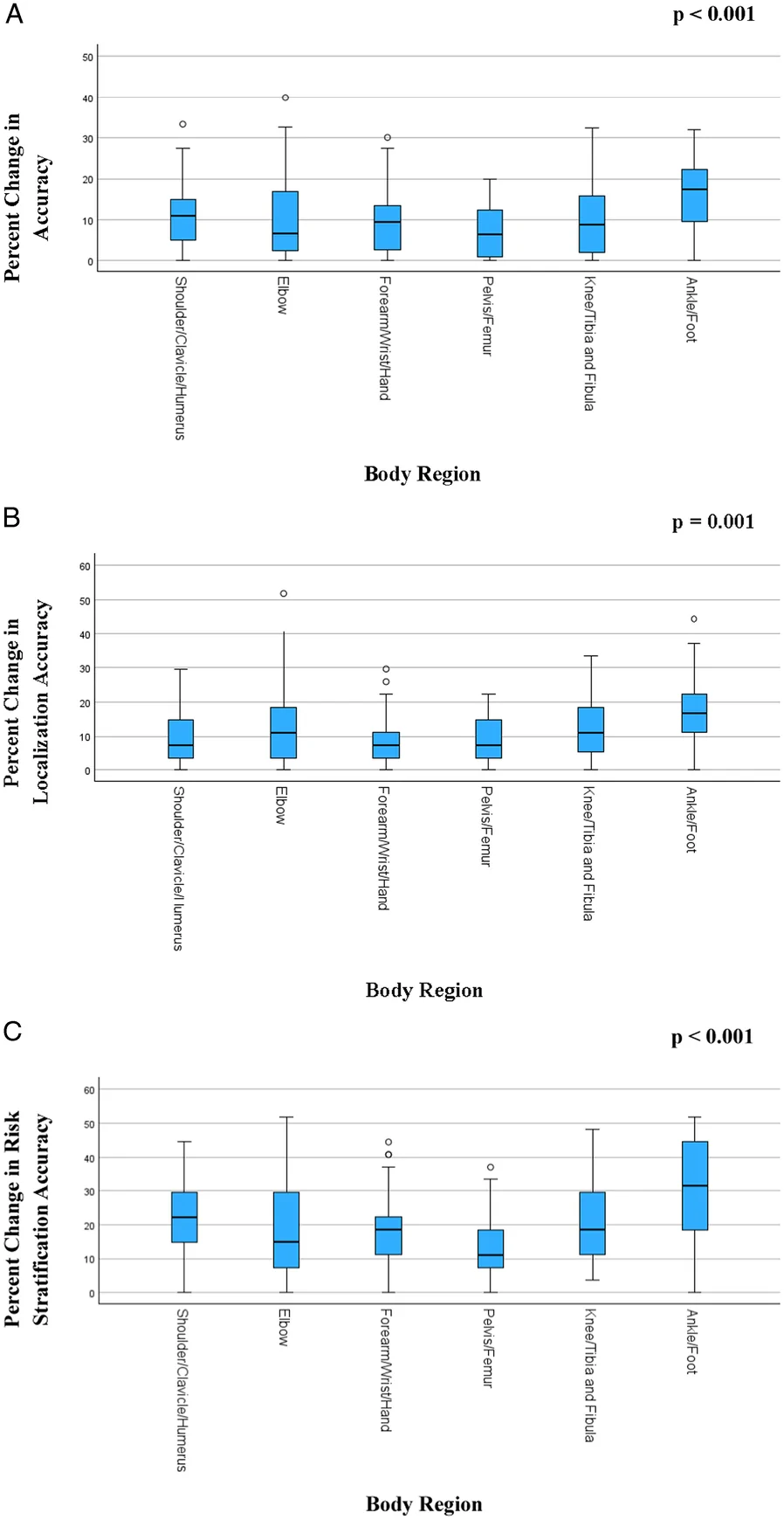
A comparison of the change in performance metrics between body regions. There was a statistical difference between body regions with respect to change in diagnostic accuracy (A) and change in accuracy for locating (B) and risk stratifying the fracture (C).

For fracture/dislocation positive cases, localization accuracy increased significantly by +11.9% (95% CI 10.7%, 13.0%) and differed between body regions (*P*=0.001) (Table 2, Fig. 3B). Post hoc adjusted analyses demonstrated increased localization accuracy gains in ankle/foot versus shoulder/clavicle/humerus (*P*=0.003) and forearm/wrist/hand (*P*<0.001). Risk stratification accuracy increased by +20.5% (95% CI: 18.9%, 22.1%) (Table 2), and also differed significantly between body regions (*P*<0.001) (Fig. 3C). Post hoc adjusted analysis demonstrated higher gains in ankle/foot versus forearm/wrist/hand (*P*=0.01), elbow (*P*=0.03), and pelvis/femur (*P*<0.001). Also, there were higher gains in shoulder/clavicle/humerus compared with pelvis/femur (*P*=0.01).

### Cases Completed to Achieve the Performance Standard

Most participants engaged in more than one body region, resulting in 246 attempts by 115 participants to reach the performance standard across the 6 body regions (Table 3). Of these, 192 (78.0%) were successful. The proportion of participants who achieved the performance standard did not differ significantly between body regions (*P*=0.17) and was not associated with postgraduate year ≥3 versus ≤2 (OR: 2.1; 95% CI: 0.6, 7.5) or Switzerland versus Canada as a training site (OR: 0.6; 95% CI: 0.2, 2.2) (Table 3). The number of cases completed to reach the performance standard was strongly skewed with moderate tail heaviness (skewness 1.7, kurtosis 3.2) (Fig. 4). The median number of cases to achieve this was 141 (IQR: 96, 313; min 58, max 909), in a median time of 34.9 minutes (IQR: 23.8, 77.6; min 14.3, max 225.0). The median number of cases completed to reach the performance standard differed significantly between body regions (*P*<0.001) (Table 3). Adjusted post hoc analyses showed that fewer cases were needed to reach the performance standard in pelvis/femur compared with ankle/foot (*P*<0.001), and elbow (*P*=0.001). Conversely, more cases were required in ankle/foot versus forearm/wrist/hand (*P*<0.001) and knee/tibia-fibula (*P*<0.001).

**TABLE 3:** Proportion of Orthopaedic Surgery Postgraduate Trainees Who Completed Cases to the Performance Standard and Median Number of Cases Completed to Reach the Performance Standard

Body region	Performance benchmark achieved, no. (%) (N=246*)	Median cases completed to achieve the performance benchmark (IQR; min, max) (N=246*)
Shoulder/clavicle/humerus	43/59 (72.9)	136 (102-218; 58, 338)
Elbow	33/45 (73.3)	166 (98-398; 58, 617)
Forearm/wrist/hand	32/39 (82.1)	136 (86-216; 58, 394)
Pelvis/femur	29/33 (87.9)	95 (71-125; 58, 427)
Knee/tibia-fibula	31/35 (88.6)	155 (115-298; 58, 342)
Ankle/foot	24/35 (68.6)	433 (341-672; 62, 909)
*P*	0.170	<0.001

* Of the 115 participating OS-PGT, most engaged in more than one region; thus, not all of the 246 data points are from different participants.

IQR indicates interquartile range; min, minimum; max, maximum.

**FIGURE 4:**
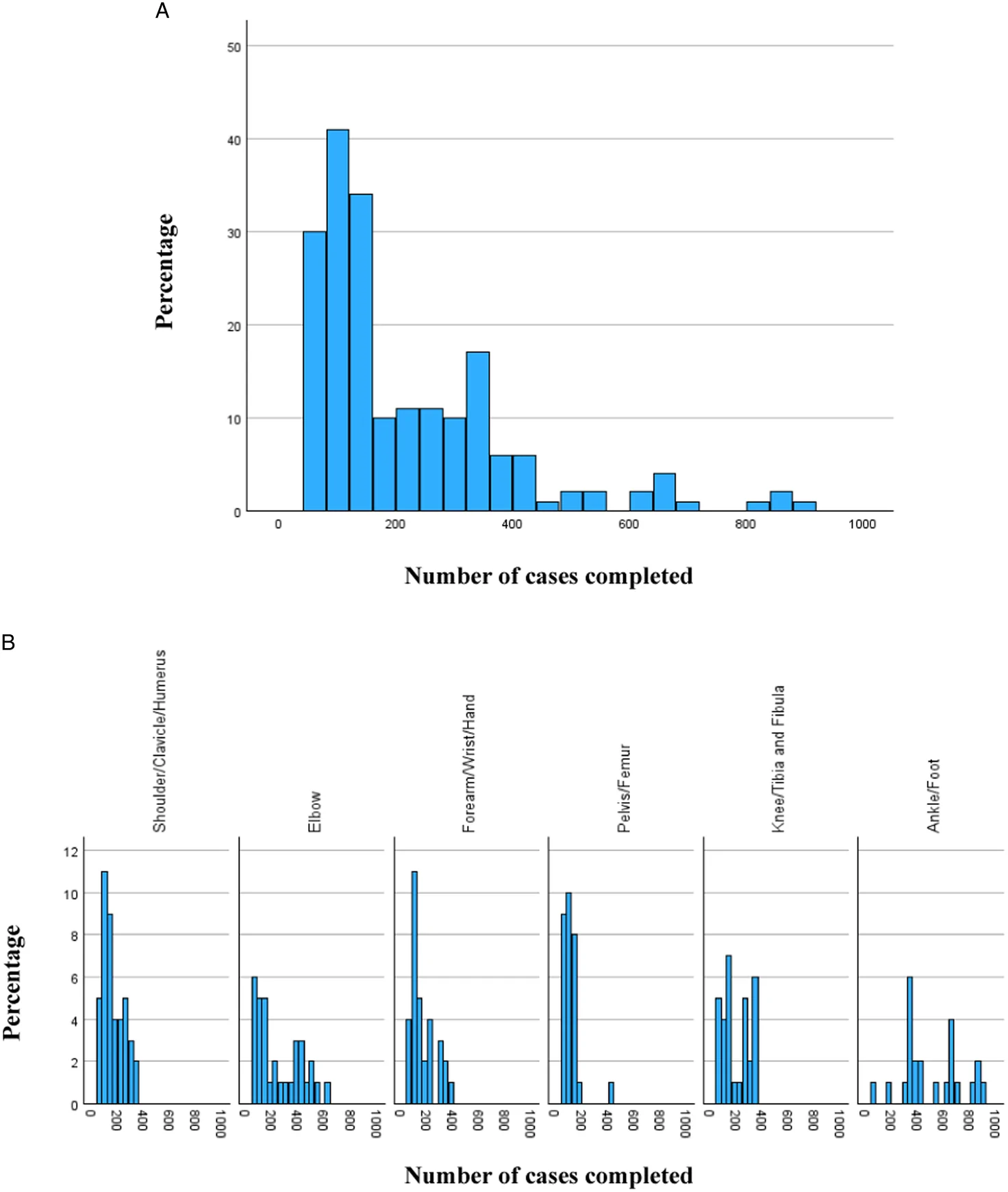
Number of cases completed to achieve the performance standard for all regions (A) and by body region (B).

### Participant Certainty

The average proportion of “definite” responses increased from 68.2% in the initial 25 cases to 89.7% in the final 25 cases (difference: 21.5%; 95% CI: 19.3%, 23.7%). Of the 51,433 “definite” responses, 3987 (7.8%) were incorrect, while of the 13,056 “probable” responses, 3088 (23.7%) were incorrect (difference: 15.9%; 95% CI: 15.1%, 16.7%). Among the 3987 incorrect “definite” responses, 2353 (59.0%) were false negatives and 1634 (41.0%) were false positives (difference: 18.0%; 95% CI: 15.8%, 20.1%). Of the 3088 incorrect “probable” responses, 1747 (56.6%) were false negatives and 1341 (43.4%) were false positives (difference: 13.2%; 95% CI: 10.7%, 15.7%).

### Participant Feedback

Of the 115 participants, 45 (39.1%) completed the post-intervention survey. Of these, 22 (48.9%) selected 90% as the preferred performance standard, 12 (26.7%) selected 80%, 5 (11.1%) selected 95%, 3 (6.7%) selected 85%, and 3 (6.7%) selected 70%. Most respondents agreed/strongly agreed that the learning objectives were met (n=42; 93.3%) and the content was relevant to their clinical practice (n=44; 97.8%). Twenty-six (57.8%) respondents agreed/strongly agreed that limited available time affected completion of the intervention. In addition, 20 (44.4%) respondents agreed/strongly agreed that the radiographic task was challenging, and 31 (68.9%) did so for the risk assignment. Despite these challenges, 33 (73.3%) agreed/strongly agreed that they were motivated to complete the intervention, and 38 (84.6%) supported it as a standard part of OS-PGT training.

## DISCUSSION

This study demonstrated that diagnostic accuracy in pMSK radiograph interpretation among OS-PGT can be significantly improved using a structured, web-based educational and assessment intervention. Large effect sizes across multiple diagnostic performance metrics indicate substantial educational impact. Accuracy gains were particularly pronounced for risk stratification in fracture/dislocation-positive cases. Most respondents considered the intervention relevant to clinical practice and supported its inclusion in orthopaedic residency training.

Participants demonstrated significant learning gains in fracture/dislocation-positive cases, with most reaching the predefined performance standard in at least one body region within 1 hour of deliberate practice. We did not detect evidence that learning gains differed by training level or country, suggesting that the intervention may be relevant across different stages of orthopaedic residency. Improved risk stratification accuracy is particularly important because it directly informs treatment urgency and type. Ankle and foot radiographs showed the greatest gains. Prior literature suggests that developmental anatomy, normal variation, and maturation-dependent pathology may contribute to interpretation challenges in this region,^38–40^ although similar challenges can occur in other pediatric musculoskeletal radiographs. Overall, these findings suggest that high-volume, performance-oriented, case-based training may help address educational challenges in this core skill for OS-PGT.

Although CBME initiatives are increasingly implemented in orthopaedic residency,^41^ defining competency and implementing practical assessment methods remain ingoing challenges.^42,43^ Our intervention addresses these challenges by providing a relatively efficient approach to improve pMSK radiograph interpretation against a predefined standard. It also offers empirical data on an attainable performance threshold and the approximate practice volume required to achieve it. Despite the modest time required to reach the standard, many respondents identified limited time as a barrier, reflecting the broader tension within CBME between increasing training requirements and clinical workload.^42,44^ Integrating the intervention into scheduled academic teaching or protected educational time may help reduce this barrier and support broader implementation. At the same time, our participants reported that they recognized the value of this type of structured training. The substantial variation in the number of cases required to achieve the performance standard suggests that learners progressed at different rates within the intervention.

The intervention also provided insight into trainee metacognitive skills. Participant certainty increased over time, and incorrect interpretations were less frequent when responses were rated as “definite.” Two-thirds of survey respondents also identified the risk stratification task as the most challenging component of the intervention, consistent with the large performance gains observed in this domain. These findings suggest that OS-PGT show some capacity for accurate self-monitoring and self-assessment.^45^ Still, false-negative errors remained more common than false positives regardless of certainty, indicating that missed injuries remain the dominant error type even when trainees feel confident. However, this finding may have been influenced by the enriched proportion of fracture-positive cases in the intervention. Nevertheless, addressing the gap between confidence and diagnostic performance may require explicit teaching on diagnostic error and cognitive bias, as well as structured feedback loops that reinforce learning from trauma decisions and complications.^46–48^

Several limitations should be acknowledged. Participation was voluntary, introducing the potential for self-selection bias. Participants may have been more motivated learners, enhancing educational outcomes, or may have had lower baseline competence in this skill area, influencing the magnitude of the observed learning effect. Consequently, the results may not be fully representative of the broader trainee population. Further, because this study used a pre-post design without a control group, the observed performance gains cannot be attributed solely to the educational content of the intervention. Potential contributors include platform familiarization, repeated exposure to the case format, and regression to the mean. Although the modest financial incentive may have increased engagement and completion rates, it was not tied to performance on individual cases and therefore was unlikely to have directly influenced the diagnostic performance estimates. The case set was enriched with fracture/dislocation-positive cases and uncommon high-risk injuries to maximize educational value, which may limit the direct applicability of performance estimates to real-world case mixes. The web-based format also lacked the clinical context available in practice. Only about one-third of participants completed the post-intervention survey, limiting the interpretation of survey findings. Although the intervention assessed performance against predefined standards, performance within the platform should not be interpreted as a comprehensive measure of clinical competence. Finally, while prior work supports that learning from this type of intervention can be retained for at least 12 months,^49^ this study did not assess whether the observed performance gains were retained over time and whether improved platform performance translates into improved clinical decision-making or patient outcomes.

There are practice implications for our results. For most OS-PGT to accumulate exposure to a diverse set of pMSK radiographs that include enough normal, normal variants, cases with a fracture or dislocation, including complex and infrequently encountered pathology, would likely take several months of exposure in a standard orthopaedic residency. In contrast, our education intervention provides this type of case exposure with just a few hours of practice. For readers interested in implementing this approach, the educational module evaluated in this study is currently available to training programs through ImageSim (https://imagesimcme.com/packages/ortho). Of note, while access to the course is fee-based, neither the authors nor the content advisors receive compensation from course sales. Future work could evaluate its implementation and effectiveness across additional institutions and learner populations.

## CONCLUSIONS

Orthopaedic surgery trainees achieved substantial improvements in pediatric musculoskeletal radiograph interpretation through a structured web-based educational and assessment intervention. The intervention improved fracture/dislocation detection, localization, and risk stratification and was well received by participants. Variation in diagnostic performance across body regions and individuals highlights the need for flexible, region-specific instructional approaches. These findings support incorporating performance-based, case-based educational tools into orthopaedic residency training to supplement existing didactic and clinical teaching.

## Supplementary Material
